# Pancreatic cancer detection with a non-contrast MR protocol: is it reliable?

**DOI:** 10.1007/s11547-023-01680-z

**Published:** 2023-07-29

**Authors:** Francesca Maio, Vincenzo Pasqualino, Luca Bertana, Silvia Venturini, Valeria Cantoni, Michele Fusaro, Giovanni Morana

**Affiliations:** 1 Department of Advanced Biomedical Sciences, Federico II University, Via Pansini 5, 80131 Naples, Italy; 2 National Cancer Institute IRCCS “Fondazione G. Pascale” Radiology Department, Via Semmola 52, 80131 Naples, Italy; 3 Department of Radiology, Padua University, Via 8 Febbraio 1848, 2, 35122 Padua, Italy; 4 Department of Radiology, General Hospital Ca’ Foncello, Piazzale dell’ Ospedale 1, 31100 Treviso, Italy

**Keywords:** Pancreatic cancer, Inter observer agreement, Patients, Pancreatic lesions, MRCP, Retrospective study

## Abstract

**Purpose:**

The pancreatic cancer (PC) is the 4th leading cancer-related death, becoming the second one by 2030, with a 5 year survival rate of 8%. Considering its increased incidence in high-risk categories compared to the general population, we aimed to validate a non-contrast MR protocol, to detect PC in its earliest phase, which could be suitable as a screening tool in high-risk patients.

**Materials and methods:**

In this retrospective study, we selected 200 patients (> 40 years) from our radiological database, which performed upper abdominal MRI between 2012 and 2017. 100 were negative for pancreatic lesions and 100 positive for pancreatic lesion (< 30 mm). The latter group included: 40 PDAC (pancreatic adenocarcinoma), 42 BD-IPMN (Branch Duct- Intraductal Papillary Mucinous Neoplasm), 10 PNET(pancreatic neuroendocrine tumor), 4 SCN(serous cystic neoplasm), 3 IPS(intrapancreatic spleen), 1 MCN(mucinous cystic neoplasm). Three readers (R1, R2 and R3) with a high, medium and low experience, respectively, analysed, first, the non-contrast MR sequences (single-shot T2w breath-hold, GE T1w FS, DWI and 2D/3D MRCP), and then the standard MR protocol, independently, randomly and anonymously. Readers identified or excluded the presence of pancreatic lesion, in both reading sessions. These results were compared with the histopathological diagnosis, and then divided into 3 different classes of lesions: all lesions, pancreatic adenocarcinoma and solid lesion. Mcnemar’s test was used to compare the results. The inter-observer agreement was determined according to the kappa statistic in both protocols, and then the inter-protocol agreement was calculated.

**Results:**

The non-contrast MR protocol has reached statistical parameters values ranging between 83% in SE (sensitivity) by R3 and 99% in NPV (negative predictive value) by R1. The standard MR protocol has reported slight increasing statistical parameters compared to those of the proposed one. However, there are not significant statistical differences between the both protocols. The proposed non-contrast MR protocol has reported the highest NPVs in the PDAC group detection (R1: 99%, R2: 99%, R3: 98%). In all groups of lesions, the agreement between the two protocols was excellent for each Reader ranging from 96 to 98%.

**Conclusion:**

The proposed non-contrast MR protocol showed high PC detection values and a time execution ≤ 20 min. Therefore, it can be proposed as a screening tool in high-risk patients.

## Introduction

Pancreatic cancer (PC) is the fourth leading cause of cancer-related death in the USA and many Western countries [1] with 47.050 deaths in 2020 in USA [2]. During 2008 and 2016, the death rate increased by 0.4% per year [2]. Although, it is predicted to become the second death leading cause by 2030 [3]. Unfortunately, the late PC diagnosis makes less than 20% of patients fit for surgery, with a 5 year survival rate of 8% [4]. On the contrary, an early diagnosis, with a PC < 10 mm without nodal involvement, carries out a significantly better prognosis, and a 5 year survival rate up to 100%, in some series [5].

Given the low incidence of PC in the general population, population-based screening is not considered cost-effective [6]. However, selected patients with increased risk, of at least 5–10 times higher than the general population, could benefit from screening. Patients with chronic pancreatitis [1], Familial pancreatic cancer (FPC) and some Inherited cancer syndromes [1, 7–9] present a raised lifetime risk of PC with an early-onset of PC in family members [10]. The high incidence of PC in these groups justifies the screening, which should be started between 40 and 50 years, and performed until the patients become “unfit for surgery” [9].

Screening program for PC appears to constitute a cost-effective intervention [11], especially for Patients with Hereditary Pancreatitis [12]. According to some Authors, EUS (endoscopic ultrasound) and MRI are the most suitable tools for pancreatic imaging in small lesions detection [13]. However, MRI is more easily accepted as a screening tool, with a better cost–benefit balance [9, 14].

Some Centers suggest a yearly interval follow-up [14–16], although this could be insufficient to detect PC in the earliest phase, and a shorter time window could be desirable, as suggested by some Authors [17]. However, the standard MR protocol is time consuming and costly. Therefore, a shorter and cheaper assessment of the pancreas with a high negative predictive value (NPV) should be desirable.

The aim of this study is comparing a non-contrast MR protocol to the standard one in the small PC detection.

## Materials and methods

As a retrospective study, patient informed consent was waived. This study has obtained Institutional Review Board approval on 20.06.2019 n. 699/CE Marca.

### Patients’ selection

In our radiological database, from January 2012 to March 2017, were found 4250 patients with upper abdominal MR study. From those, using the clinical folders, radiology resident “in training” selected 1365 patients following these criteria:

- Age > 40 years,
- Without history of previous abdominal surgery either gastro-intestinal intervention or chemo-radio-therapy for upper abdominal tumours
- Availability of a complete MR study, including pre- and post-contrastographic sequences DWI (Diffusion Waited Images) and MRCP (Magnetic Resonance Cholangiopancreatography).

Then, using the radiological reports, were selected only patients with:

- PC smaller than 30mm,
- Solid lesions, not PC, < 30 mm
- Cystic pancreatic lesions < 30 mm
- Patients without pancreatic lesions, who had performed MR imaging for other conditions, without evidence of a solid mass elsewhere in the field of study.

The resulting pool of 658 patients was included. Then, it was divided in two parts: one containing patients with pancreatic lesions (387), one those without pancreatic lesions (271). 100 patients were selected in each group, randomly. The patients’ recruitment was interrupted when the predefined number was reached, in order to recreate the different conditions found in patients at high-risk to develop a PC [9, 18].

The main focus of a screening test is to find lesions in their early stages; therefore, we chose the threshold of < 30 mm for pancreatic lesions, in order to exclude cystic lesions > 30 mm, considered as a worrisome feature [19]. As a retrospective study, the patients’ clinical folders did not contain adequate information allowing Authors to select only patients belonging to a poll of high-risk people for PC. However, patients older than 40 yrs have been selected, in order to analyse a pancreatic parenchyma as similar as possible to the high-risk ones [9].

#### Lesions’ groups characteristics

Two groups containing 100 patients, each, resulted after the reported above patients’ selection. The one with pancreatic lesions included: 40 pancreatic adenocarcinoma (PDAC), 42 Branch Duct- Intraductal Papillary Mucinous Neoplasm (BD-IPMN), 10 pancreatic neuroendocrine tumor (PNET), 4 serous cystic neoplasm (SCN), 3 intrapancreatic spleen (IPS), 1 mucinous cystic neoplasm (MCN). Their localization into the pancreatic parenchyma was: 37 in the head, 34 in the body, 19 in the tail, 10 were multifocal (IPMN).

The other group contained 100 patients without pancreatic or abdominal disease.

#### Diagnosis confirmation

Pathology evaluation of surgically resected served as a standard of reference for the diagnosis of PDACs (n = 40). About PNETs, 6 were resected, while 4 had somatostatin analogue nuclear medicine study confirmation, and followed-up due to the small size. IPSs were characterized by Nuclear Medicine. For cystic lesions, benignity was confirmed by at least 2 years follow-up.

A Radiologist, expert in abdominal MRI and pancreatic pathologies, revised the MRI exams of the negative patients group, to confirm that no abdominal or pancreatic disease were present.

### Imaging protocol

MR studies were performed with a 1.5 T MR machine (Magneton Avanto, Siemens Healthineers, Forchheim, Germany). The standard MR protocol for a complete workup of the pancreas requires single-shot T2w breath-hold (SSFSE T2w BH) on axial and coronal plans, axial Gradient echo T1w with fat saturation (FS-GRE T1w), axial “in–out phase” T1w, axial DWI with 4 b values (0–50-400–800), 2D/3D MRCP and a gadolinium-enhanced axial 3D GRE multiphase acquisition [20, 21].

For our non-contrast MR protocol, we selected the following sequences from the standard protocol: axial and coronal SSFSE T2w BH, axial FS-GRE T1w, DWI (2 b values 50–800), 2D/3D MRCP (Table 1).

**Table 1:** MRI study protocols

Standard MR protocol sequences	Proposed MR protocol sequences	MR protocol parameters	Acquisition time
SSFSE T2w BH	SSFSE T2w BH	**Plan:**axial	42ʺ
		**FoV**: 380 mm	
		**FoV phase**: 81.3%	
		**Slice thickness**: 6 mm	
		**TE:** 92 ms	
		**TR:**1000.0 ms	
SSFSE T2w BH	SSFSE T2w BH	**Plan:** coronal	38ʺ
		**FoV**: 400 mm	
		**FoV phase**: 100.0%	
		**Slice thickness**: 5 mm	
		**TE:** 90 ms	
		**TR:**1000.0 ms	
T2w BLADE FS trig		**Plan:**axial	2′40ʺ
		**FoV**: 380 mm	
		**FoV phase**: 100.0%	
		**Slice thikness**: 6 mm	
		**TE:** 89 ms	
		**TR:**2200.0 ms	
DWI 0–50-400–800 (6 average)	DWI 50–800 (6 averages)	**Plan:**axial	3′47ʺ
		**FoV**: 380 mm	
		**FoV phase**: 100.0%	
		**Slice thikness**: 4 mm	
		**TE:** 61 ms	
		**TR:**4600.0 ms	
GRE T1w IN/OUT		**Plan:**axial	32ʺ
		**FoV**: 380 mm	
		**FoV phase**: 78.1%	
		**Slice thickness**: 6 mm	
		**TE:** 2.38 ms	
		**TR:**137.0 ms	
GRE T1w FS	GRE T1w FS	**Plan:**axial	40ʺ
		**FoV** 380 mm	
		**FoV phase**: 84.4%	
		**Slice thkness**: 6 mm	
		**TE:** 3.55 ms	
		**TR:**125.0 ms	
2D BH MRCP	2D BH MRCP	**FoV**: 400 mm	5ʺ
		**FoV phase**: 100.0%	
		**Slice thickness**: 6.0 mm	
		**TE:** 1.8 ms	
		**TR:**745.70 ms	
3D RT MRCP	3D RT MRCP	**FoV**: 380 mm	5′–10′
		**FoV phase**: 100.0%	
		**Slice thickness**: 1.5 mm	
		**TE:** 680 ms	
		**TR:**1700.0 ms	
Gd-enh 3D GRE T1w FS		**Plan:**axial/coronal	
		**FoV**: 370 mm	
		**FoV phase**: 81.3%	
		**Slice thickness**: 2.5 mm	
		**TE:** 3.47 ms	
		**TR:**1.27 ms	
	Total	in/out room	≤ 20′

^i^*SSFSE T2w BH* single-shot T2w breath-hold, *T2w RADIAL FS* T2w radial fat saturated sequences, *DWI* diffusion waited imaging, *GRE T1w IN/OUT* gradient echo T1w in and out phase, *2D BH MRCP* bi-dimensional Breath hold MR cholangiopancreatography, *3D RT MRCP* three-dimensional respiratory trigged MR cholangiopancreatography

### Imaging analysis

3 Readers, with different level of experience in MRI of the pancreas (R1: > 15 yrs; R2: > 5 yrs; R3: “in–training”) independently and randomly, reviewed the cases anonymously, unaware of symptoms and clinical history of the patients. Readers were aware that solid, cystic or no pancreatic lesions could be observed.

Image analysis has made by using the workstation of our PACS system (Fuji Synapse, version 4.1.6.0, Fujifilm, Valhalla, NY, USA).

The radiology resident “in-training” was responsible for the management of cases review, showing only the chosen sequences for the analysis. In patients with multiple studies, the baseline MR study was chosen for the reading session. The assessment of the images quality was not conducted before the reading session, as well as to recreate as much as possible a realistic situation.

Reading session was performed in two steps: first, the Readers analysed the proposed non-contrast MR protocol; secondly, one month later, with a new random order, they analysed the standard MR protocol.

**Fig. 1:**
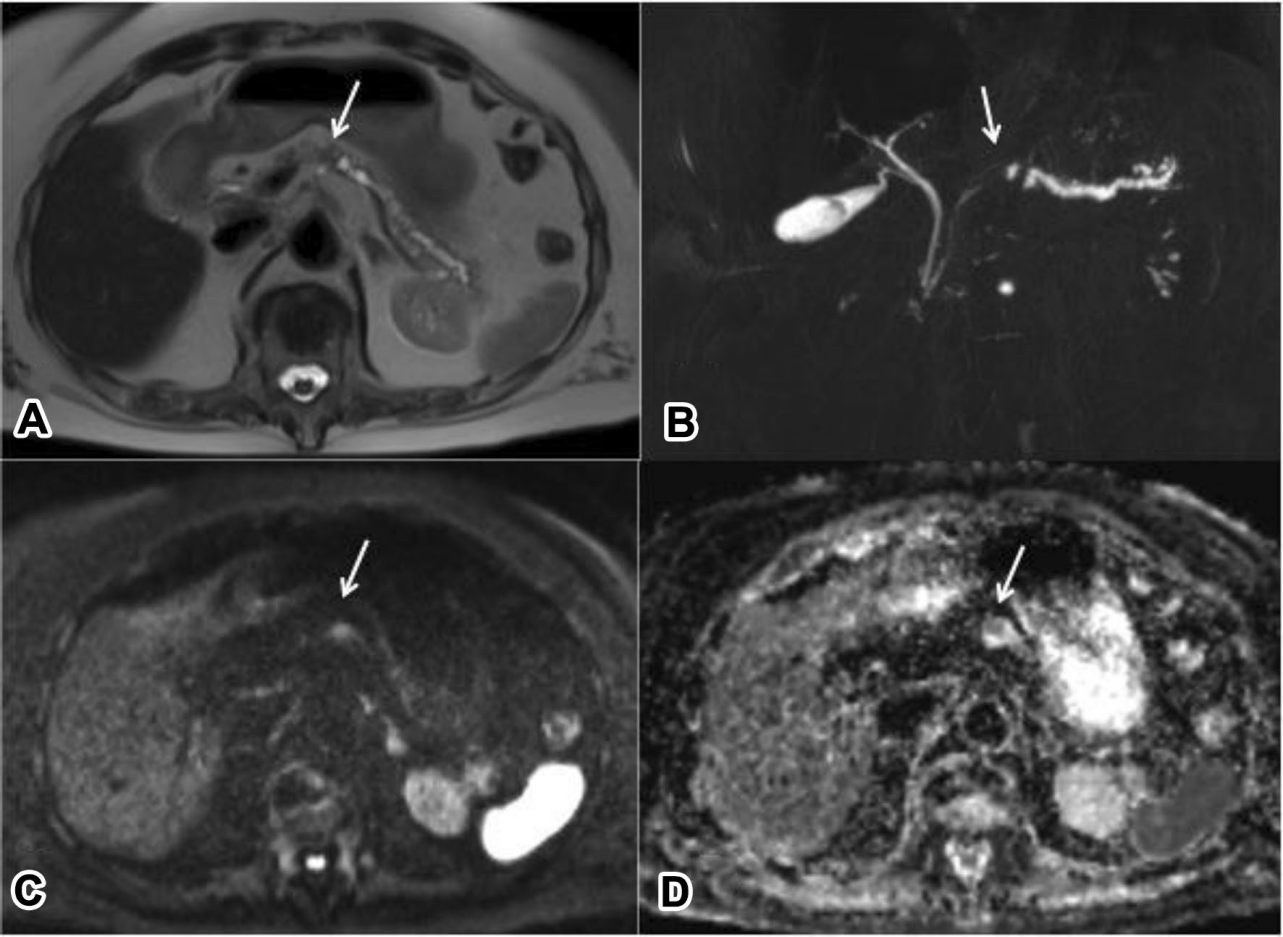
Small PDAC. At T2 HASTE (**a**) a moderate dilatation of mpd can be appreciated, with a stenosis at the body level (arrow). At MRCP (**b**) the stenosis of mpd (arrow) and its dilatation upstream is confirmed. At DWI (b 800) a small hyperintence spot is appreciable at the level of stenosis (arrow in C), which appears hypointense on ADC map (arrow in D)

The presence of pancreatic solid lesion was based either on a discernible mass-like lesion in the pancreatic parenchyma or based on secondary features. Those are: focal main pancreatic duct (MPD) dilatation, focal stenosis of MPD, even if without upper dilation, loss of regular pancreatic lobulations, dilatation of choledochus, focal hypointensity on FS- GRE T1w [22], focal hyperintensity on high b values DWI or hypovascular lesion during dynamic enhancement (Fig. 1).

Pancreatic cystic lesions were evaluated following the International consensus guidelines [19]: BD-IPMN as a cystic lesion > 5 mm communicating with MPD on MRCP, while MCNs and SCN were evaluated by morphological appearance (oligocystic for MCN, microcystic for SCN) [19].

Based on these criteria, in each reading session, Readers indicated whether or not a pancreatic lesion is present, suggesting the solid or cystic nature.

### Statistical analysis

Continuous variables are expressed as mean ± SD, and categorical data as frequencies or percentage.

In each reading session and for each Reader, sensitivity (SE), specificity (SP), positive predictive value (PPV) and NPV were calculated for pancreatic lesions detection.

Results of both reading sessions were compared with the final diagnosis according to three different classes of lesion: *solid lesions, PDAC* and *all lesions*; Mcnemar’s test was used to compare the results of the protocol in the different classes of lesions between Readers. Inter-protocol agreement for each Reader was determined according to the kappa statistic, for the identification of pancreatic lesions according to the three different classes of lesions. Moreover, the inter-reader agreement was calculated on a three-category (R1 vs. R2 vs. R3) basis, in both protocols. Poor, moderate, good and excellent agreement are indicated by κ values ≤ 0.40, from 0.41 to 0.60, from 0.61 to 0.80 and > 0.80, respectively. A *p* value < 0.05 was considered as statistically significant. Statistical analysis was performed with a commercially available software package (SPSS, version 20.0 for Windows, SPSS Inc., Chicago, IL, USA) and STATA 14 software (StataCorp, College Station,TX).

## Results

No statistical differences were observed in the age of the two groups of patients (Patients with pancreatic lesions: mean 67.6 ± 10 years; Patients without pancreatic lesions: mean 64.8 ± 10 years). The mean size of the pancreatic lesions group was 15.6 mm ± 6.045 mm.

Results of reading sessions are reported on Table 2. On Table 3 are reported SE, SP, PPV and NPV of different Readers in the different classes of lesions, in both MR protocols. On Table 4 is shown the inter-protocol agreement for each Reader. Finally, on Table 5 is illustrated the agreement between the Readers.

**Table 2:** Reading results Focused on solid pancreatic lesion detection in both protocols

Diagnosis	Total	Proposed protocol			Standard protocol		
		R1	R2	R3	R1	R2	R3
PDAC	40	39	39	37	40	38	39
IPMN	42	0	1	1	0	1	0
PNET	10	5	2	1	1	1	1
Other lesions	8	2	2	1	0	0	1
Negatives	100	0	1	1	0	1	2

^i^*PDAC* adenocarcinoma, *IPMN* intraductal papillary mucinous neoplasm, *PNET* pancreatic neuroendocrine tumor, *R*1 Reader > 15 year experience, *R*2 Reader > 5 years experience, *R*3 fellow

**Table 3:** Reading session statistical analysis in both protocols

	Proposed protocol				Standard protocol				*p* value			
	SE	SP	PPV	NPV	SE	SP	PPV	NPV	SE	SP	PPV	NPV
*All lesions*												
R1	96%	98%	98%	96%	98%	100%	100%	98%	0.68	0.50	0.50	0.68
	(92–98%)	(94–99%)	(94–99%)	(92–98%)	(94–99%)	(97–100%)	(97–100%)	(94–99%)				
R2	94%	94%	94%	94%	97%	95%	95%	97%	0.50	0.99	0.99	0.50
	(89–96%)	(89–96%)	(89–96%)	(89–96%)	(93–98%)	(90–97%)	(90–97%)	(93–98%)				
R3	94%	98%	97%	94%	95%	98%	97%	95%	0.99	0.99	0.99	0.99
	(89–96%)	(94–99%)	(94–99%)	(89–96%)	(90–97%)	(94–99%)	(94–99%)	(90–97%)				
*PDAC*												
R1	97%	95%	84%	99%	100%	99%	97%	100%	0.99	0.07	0.07	0.99
	(93–99%)	(91–97%)	(78–89%)	(96–99%)	(97–100%)	(96–99%)	(94–99%)	(97–100%)				
R2	97%	96%	86%	99%	95%	98%	92%	98%	0.99	0.50	0.50	0.99
	(93–99%)	(92–98%)	(81–90%)	(96–99%)	(90–97%)	(94–99%)	(87–95%)	(95–99%)				
R3	92%	97%	90%	98%	97%	97%	90%	99%	0.62	0.99	0.99	0.62
	(87–95%)	(93–99%)	(85–93%)	(94–99%)	(93–99%)	(93–99%)	(85–94%)	(96–99%)				
*Solid lesions*												
R1	92%	98%	96%	97%	96%	100%	100%	98%	0.68	0.50	0.50	0.68
	(87–95%)	(95–99%)	(92–98%)	(93–99%)	(92–98%)	(97–100%)	(97–100%)	(95–99%)				
R2	84%	98%	95%	94%	90%	97%	92%	96%	0.58	0.68	0.68	0.58
	(79–89%)	(95–99%)	(91–98%)	(90–97%)	(85–94%)	(93–98%)	(87–95%)	(92–98%)				
R3	83%	98%	93%	94%	88%	98%	94%	96%	0.60	0.99	0.99	0.60
	(76–87%)	(94–99%)	(89–96%)	(89–96%)	(83–92%)	(94–99%)	(89–96%)	(90–97%)				

^i^*PDAC* adenocarcinoma, *SE* sensitivity, *SP* specificity, *PPV* positive predictive value, *NPV* negative predictive value. *R*1: Reader > 15 years experience, *R*2: Reader > 5 years experience, *R*3: fellow

**Table 4:** Inter-protocol agreement in all different group of lesion detection

PP versus SP	*k*	95% CI	Agreement (%)
*All lesions*			
R1	0.96	0.92–0.99	98
R2	0.96	0.92–0.99	98
R3	0.97	0.93–0.99	98
*PDAC*			
R1	0.89	0.82–0.97	96
R2	0.91	0.84–0.98	97
R3	0.91	0.83–0.91	97
*Solid lesions*			
R1	0.94	0.89–0.99	98
R2	0.93	0.87–0.99	97
R3	0.93	0.87–0.99	97

^i^*PP* Proposed Protocol, *SP* standard protocol, *PDAC* adenocarcinoma, *R*1: Reader > 15 years experience, *R*2: Reader > 5 years experience, *R*3: fellow, *K*: kappa statistic

**Table 5:** Inter-Readers agreement in pancreatic lesion detection in both protocols

	*R* relation	*k*	95% CI	Agreement (%)
*All lesions*				
Proposed protocol	R1/R2	0.88	0.81–0.94	94
	R1/R3	0.87	0.80–0.93	94
	R2/R3	0.84	0.76–0.91	92
	MEAN	0.86	0.79–0.92	
Standard protocol	R1/R2	0.92	0.86–0.97	96
	R1/R3	0.91	0.85–0.96	95
	R2/R3	0.85	0.77–0.92	92
	MEAN	0.89	0.82–0.95	
*PDAC*				
Proposed protocol	R1/R2	0.87	0.79–0.95	95
	R1/R3	0.83	0.74–0.93	94
	R2/R3	0.82	0.72–0.91	94
	MEAN	0.84	0.75–0.93	
Standard protocol	R1/R2	0.90	0.83–0.98	97
	R1/R3	0.91	0.85–0.99	97
	R2/R3	0.87	0.78–0.95	95
	MEAN	0.89	0.82–0.97	
*Solid lesions*				
Proposed protocol	R1/R2	0.89	0.81–0.96	96
	R1/R3	0.83	0.74–0.92	94
	R2/R3	0.86	0.77–0.84	95
	MEAN	0.86	0.77–0.90	
Standard protocol	R1/R2	0.90	0.84–0.97	96
	R1/R3	0.90	0.84–0.97	96
	R2/R3	0.89	0.82–0.96	96
	MEAN	0.89	0.83–0.96	

^i^*PDAC* adenocarcinoma, *R*1 Reader > 15 years’ experience, *R*2 Reader > 5 years’ experience, *R*3 fellow, *K* kappa statistic

On Table 3, all Readers reached very high values in all statistical parameters in all classes of lesions, in both protocols. In particular, in the proposed MR protocol, the statistical parameters values ranged between 83% in SE by R3 and 99% in NPV by R1. All Readers have reported a slight increase of all statistical parameters in the standard MR protocol compared to those of the proposed one. However, there are not significant statistical differences between both protocols, in the three categories of lesions, for each Reader. Furthermore, the proposed MR protocol has reported the highest NPVs in the *PDAC* group (R1: 99%, R2: 99%, R3: 98%). Moreover, in the *PDAC* identification, R2 has shown higher SE and NPVs in the proposed MR protocol compared to those of the standard one (SE: 97% vs. 95%, NPV: 99% vs. 98%). Similarly, R2 has reported higher SP and PPVs in *solid lesion* group in the proposed MR protocol compared to those of the standard one (SP: 98% vs. 97%, PPV: 95% vs. 92%). None of those differences were statistically significant. Moreover, in *solid lesions* group, R2 and R3 show lower SE and NPV results compared to those of R1, in both protocols, according to their different experience.

In both protocols, all Readers reached lower SE values in the *solid lesions* group compared to those of *PDAC* and *all pancreatic lesions* group. These results are mostly related to the poor PNET detection in the reading session (Table 2).

On Table 4, in all groups of lesions, the agreement between the two protocols was excellent for each Reader ranging from 96 to 98%.

On Table 5, an excellent agreement was found between the three Readers in both protocols. In particular, the k statistic mean results were in the proposed and standard MR protocol: *all lesions* detection as of 0.86 and 0.89, *PDAC* detection as of 0.84 and 0.89, and *solid lesions* detection as of 0.86 and 0.89, respectively.

Cystic lesions were used as a confounding factor in the pancreatic solid lesion detection, especially for the *PDAC*, therefore, no statistical results were reported for those lesions.

## Discussion

Our results show high values in all analysed parameters, especially SE and NPV, in both protocols.

No statistical differences were found between the two protocols either in the Readers capability to detect pancreatic lesions or in their agreement.

According to these results, especially the high SE and NPV values, our short protocol can be suitable in a high-risk patients’ screening program [23], thanks to the excellent results in detecting PDAC, the most frequent malignant PC [24].

Our imaging protocol was performed with a standard 1.5 T MR machine, easily available in the Hospitals. However, even if not significant statistical differences were found between Readers, is to underline that our Hospital is the second reference centre for pancreatic diseases in a large regional area of about 5 millions inhabitants, taking care of hundreds of patients with pancreatic pathologies and more than 70 pancreatic resections yearly. Thus, our results suggest that an experienced Radiologist in pancreatic imaging should perform the reading session.

The proposed non-contrast MR protocol does not require a long acquisition time (about 20 min in/out room) and the use of contrast media, avoiding gadolinium brain deposition [25].

MRI has an high sensitivity to identify solid pancreatic lesions, thanks to the high contrast between normal parenchyma and pancreatic lesions in FS-GRE T1w [22] and DWI images [21]. Although DWI images can have some limitations in small pancreatic solid lesions detection [26], the combination of the different sequences allowed us to have the reported excellent results. Consequently, it is highly recommended as screening modality for PC.

According to some screening program experience [11–14], growth rate is correlated to the survival outcome. Mean volume doubling time (VDT) of pancreatic cancer ranges from 132,3 days [27] to 144 days [28], although in Ahn et Al. it varied from less than one month to more than four years [27]. Moreover, Mortenson et Al. observed that the surface area of a PC may grow yearly from 1.17 to 7.15 times [17], suggesting a 3 months follow-up interval. A recent study, based on genomic sequencing on cancer cells in seven PC patients, reports an average of 11.7 years elapsed from tumor initiation to overt cancer development and an average of 6.8 years elapsed between the development of overt cancer and the development of metastatic disease. Thus, there is a large time window for detection of PC while the disease is in its earliest and treatable stages [29].

**Fig. 2:**
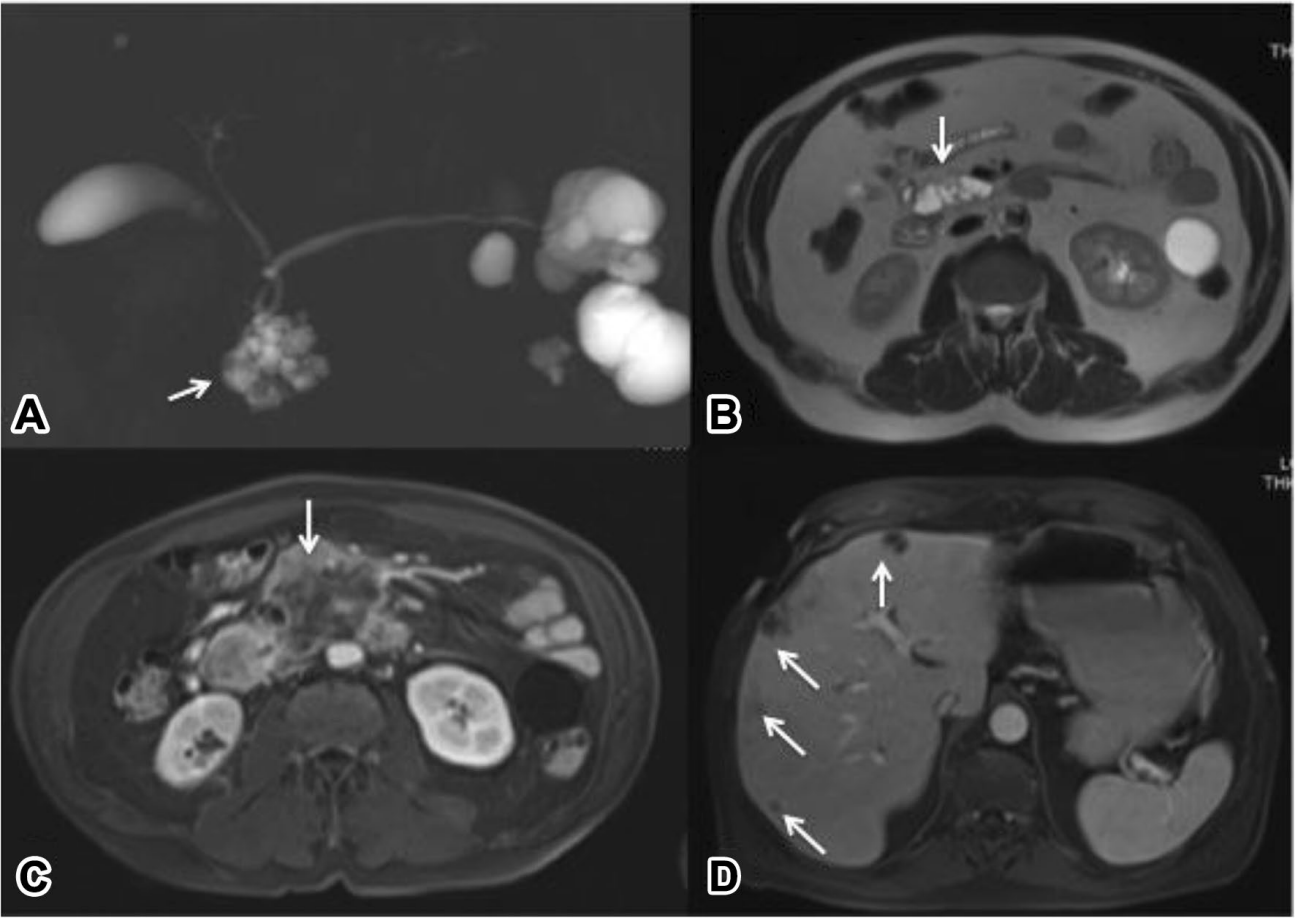
Degenerated IPMN. At MRCP a BD-IPMN is appreciable in the uncinate process. The lesion does not show solid nodules at T2 HASTE, as well as at DWI and contrast-enhanced MRI (not shown). Even after EUS no worrisome features were appreciated. At follow-up after 10 months a large hypovascular lesion occupies the uncinate process (arrow: C), with multiple hepatic metastases (arrows: D)

However, the predicted progression rate suggests that once a PC is detectable by diagnostic tests (stage I), its growth and progression to stage IV disease can occur within 1 year [30]. Based on these observations, 1 year screening program interval time can be insufficient to diagnose the PC in the early and resectable stage (Fig. 2). For this reason, a short and, relatively, cheap imaging test is requested for high-risk patients, allowing them to be screened twice a year. Moreover, a non-contrast MR protocol avoids the spending time to read and explain to the patients the contrast media informed consent, the vein cannulation and the post-procedure activities, reducing also any contrast-media adverse reaction and the care-time of the patient.

**Fig. 3:**
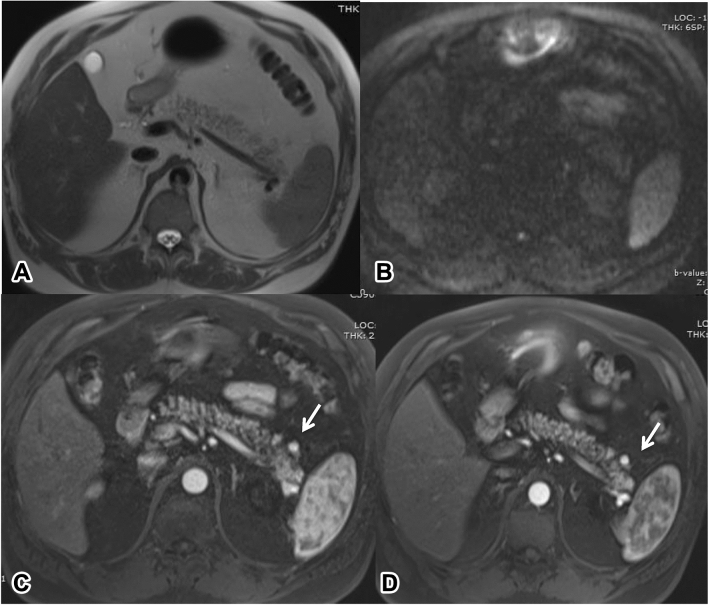
**A**–**D**: small PNET (< 10 mm). On T2 HASTE (**A**), and DWI (b 800: **B**), no lesion with pathologic features is appreciable. In the complete workout, a small hypervascular lesion is appreciable during arterial phase (**C**) after Gadobenate Dimeglumine (Multihance, Milano, Italy). The lesion is unchanged in comparison to a previous study, 1 year before (**D**)

About the PNET results, according to WHO classification 2010 [31] and Kuo et Al. [32] in PNETs ≤ 2 cm the most significant predictors of disease-specific survival are grading and growth. Although SEER database excludes PNETs to be considered benign, and small size does not preclude malignant behaviour [32], Bettini et al. found that out of 51 R0 resected incidental non functional PNETs (NF-PNETs) ≤ 2 cm, only 6% were malignant and in the long term follow-up (median 47 months) there were no deaths directly related/caused by the specific disease [33]. It is also reported that there is no direct benefit in early surgery approach for PNETs ≤ 2 cm [34]. So in a systematic screening programme, it is possible to find in a further follow-up study small missed PNETs without affecting long-term patient’s prognosis (Fig. 3) [35–37]*.*

Furthermore, in patients with cystic lesions or cystic PNETs, DWI is the sequence for suspecting malignant degeneration: DWI is helpful for distinguishing mucin plug from solid nodule in cystic lesions [38], but mostly there is a significant correlation between diffusion restriction and invasiveness/malignancy, as demonstrated by Kim [39]. The most “time consuming” sequence is 3D MRCP, which, due to the triggered acquisition, can last several minutes in uncooperative patients. Some studies have demonstrated that in the visualization of pancreatic ductal system there are not significant differences between 2 and 3D MRCP [40, 41], thus, it can be concluded that a 2D SSFSE is a suitable sequence in a short pancreatic screening protocol. Recent development of compressed sensing MRCP allow to obtain 3D MRCP in a single breath hold, thus reducing the time requested [42].

Our study has several limitations, as a retrospective design, might have a possible selection bias, which we have tried to overcome making a randomized selection of cases including a large variability of pancreatic alterations. The selection of all cases comes from a radiology database, which does not include cases that could have been positive on EUS or other non-radiologic workups. The cohort of patients is not a high-risk PC category, however, only patients over 40 yrs were included, trying to replicate similar pancreatic parenchyma morphology. The only group which is not comparable is the Hereditary Pancreatitis group, where early advanced alteration of pancreatic parenchyma and ductal system are associated in a very young population [43]. In this group, a specific prospective study should be conducted. In this last group of high-risk patients some parenchymal alterations, due to chronic inflammation, such as focal stenosis of MPD or loss of regular pancreatic lobulations, could simulate a pancreatic lesion. As well as, patients with chronic pancreatitis could present the same pancreatic alterations described above, with a very challenging capability to differentiate suspect lesions from chronic alterations. Consequently, the number of patients recalled for a complete work-up could be higher than needed, thus we think it is necessary to perform the MRI in the same center, where previous exams are easily available and comparable, allowing the detection of minimal changes in the different studies.

Even if the PDACs included in our study were resectable, some small PDACs in a screening scenario could face locally advanced or metastatic, which means that they would not be considered as resectable before neo-adjuvant chemotherapy. However, thanks to the short acquisition time of the proposed non-contrast MR protocol, it can be offered twice a year, reducing the risk to detect a PDAC in an advanced stage. In comparison to EUS, MRI has the advantage of a whole exploration of the upper abdomen, allowing in the same setting the detection or exclusion of small liver metastases, especially thanks to DWI [44–50].

Although the most experienced radiologist (R1) had excellent results (SE 97%, SP 95%), even the less experienced radiologist (R3) obtained satisfactory results (SE 92%, SP 97%). Considering as standard of reference the breast cancer screening double reading which reports sensitivity of 72% and specificity of 97% [51, 52], even if in a different situation, which is not a screening ones, those achieved results seam to be quite high and encouraging for PC detection.

## Conclusion

The reported high PC detection values, the short time consuming (less than 20 min “in and out door”) and the “free gadolinium injection”, makes our proposed non-contrast MR protocol suitable to be used as a screening tool in high-risk patients, reducing the time lapse between the exams, with more chances to the patient to be diagnosed in the early phase.

## Data Availability

The authors confirm that the data supporting the findings of this study are available within the article and its supplementary materials.

## Abbreviations

PC: Pancreatic cancer
BD-IPMN: Branch duct-intraductal papillary mucinous neoplasm
FPC: Familial pancreatic cancer
HP: Hereditary pancreatitis
PDAC: Pancreatic adenocarcinoma
PNET: Pancreatic neuroendocrine tumor
MPD: Main pancreatic duct
IPS: Intrapancreatic spleen
MCN: Mucinous cystic neoplasm
SCN: Serous cystic neoplasm
SE: Sensitivity
SP: Specificity
NPV: Negative predictive value
PPV: Positive predictive value
VDT: Volume doubling time
EUS: Endoscopic ultrasound
DWI: Diffusion waited images
MRCP: Magnetic resonance cholangiopancreatography
SSFSE T2w BH: Single-shot T2w breath-hold
FS-GRE T1w: Gradient Echo T1w with fat saturation
